# P-1602. Urinalysis with Reflex Urine Culture to Screen for Asymptomatic Bacteriuria in Kidney Transplant Recipients: Opportunities for Diagnostic and Antimicrobial Stewardship

**DOI:** 10.1093/ofid/ofae631.1769

**Published:** 2025-01-29

**Authors:** Hayato Mitaka, Evan Clemens, Eleanor Oken, Robert M Rakita, Ajit P Limaye, Ralph Tayyar

**Affiliations:** University of Colorado, Aurora, Colorado; University of Washington, Seattle, Washington; University of Washington, Seattle, Washington; University of Washington, Seattle, Washington; University of Washington, Seattle, Washington; University of Washington, Seattle, Washington

## Abstract

**Background:**

Routine screening and treatment of asymptomatic bacteriuria (ASB) in kidney transplant recipients (KTRs) beyond 1 month post-transplant are no longer recommended due to potential harm. Evidence from clinical trials demonstrated that screening and treatment of ASB does not reduce the risk of pyelonephritis, acute rejection or long-term graft impairment. However, ASB screening post-transplant remains a common practice. This study characterizes our institution’s current practice with associated testing costs regarding screening and treatment for ASB for the first 3 months post-transplant.

Estimated annual cost of urinalysis and urine culture in kidney transplant recipients at University of Washington Medical Center

**Figure:**
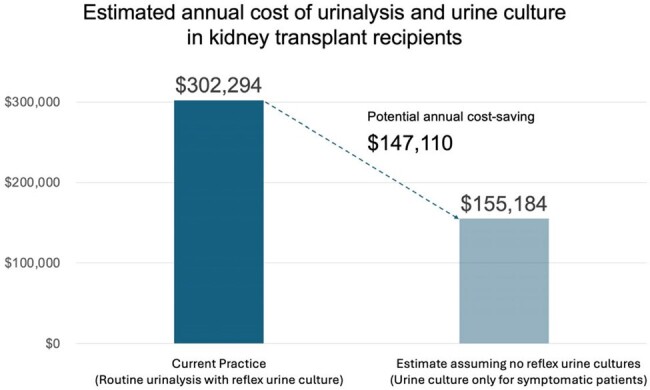

The estimated annual cost of urinalysis and urine culture was calculated based on the testing cost in 50 patients for the first three months post-transplant, assuming a total of 200 patients receive kidney transplant annually at our institution. The average cost of urinalysis and urine culture per person for the first three months after transplant was $1511.47. This estimate does not take into account urinalyses and urine cultures beyond three months. The estimated cost savings assume that urinalysis with reflex urine cultures is de-implemented and urine cultures are ordered only for symptomatic patients. This estimate does not consider the potential reduction in the number of urine cultures based on negative urinalysis in symptomatic patients.

**Methods:**

We conducted a retrospective review of electronic records for 50 consecutive adult KTRs at University of Washington Medical Center between January and April 2023. Data collected included demographics, laboratory results, microbiological findings, antibiotic usage for 3 months post-transplant, and rejection within 1 year post-transplant. A chart review by 2 independent reviewers was performed to determine whether each positive urine culture (UCx) represents ASB or urinary tract infection (UTI).

**Results:**

Among 50 KTRs, a total of 709 urinalyses (UA, mean 14.2 per person) and 263 UCx (mean 5.2 per person) were performed within 3 months post-transplant. Reflex from UA accounted for 95.4% of UCxs (251 out of 263), and 35.4% of UA were reflexed to UCx (251 out of 709). Of 40 positive urine cultures, 6 were considered symptomatic UTI and 34 as ASB. The mean cost of routine screening with UA with reflex UCx for the first 3 months after transplant was $1511.47. The estimated annual cost based on 200 KTRs per year exceeds $300,000 (Figure 1). All 6 UTI episodes and 5 of 34 ASB episodes (14.7%) were treated with antibiotics. Acute rejection by 1 year post-transplant occurred in 2 out of 50 patients (4%), but neither had ASB nor UTI within 3 months of transplant.

**Conclusion:**

Routine UA with reflex UCx during post-transplant clinic visits accounted for the majority of cases of ASB within the first 3 months post-transplant. Decreasing the ordering of UA-guided reflexive UCx has the potential to reduce the identification of ASB and associated costs.

**Disclosures:**

**Ajit P. Limaye, MD**, GSK: Advisor/Consultant|Memo: Advisor/Consultant|merck: Advisor/Consultant|merck: Grant/Research Support|Moderna: Advisor/Consultant|NobelPharma: DSMB member|Novartis: DSMB member|syneos: DSMB member

